# Dual-Specificity Phosphatase 1 and Tristetraprolin Cooperate To Regulate Macrophage Responses to Lipopolysaccharide

**DOI:** 10.4049/jimmunol.1402830

**Published:** 2015-05-27

**Authors:** Tim Smallie, Ewan A. Ross, Alaina J. Ammit, Helen E. Cunliffe, Tina Tang, Dalya R. Rosner, Michael L. Ridley, Christopher D. Buckley, Jeremy Saklatvala, Jonathan L. Dean, Andrew R. Clark

**Affiliations:** * School of Immunity and Infection, College of Medical and Dental Sciences, University of Birmingham, Birmingham B15 2TT, United Kingdom;; † Faculty of Pharmacy, The University of Sydney, New South Wales 2006, Australia; and; ‡ Kennedy Institute of Rheumatology, Nuffield Department of Orthopaedics, Rheumatology and Musculoskeletal Sciences, University of Oxford, Oxford OX3 7FY, United Kingdom

## Abstract

Dual-specificity phosphatase (DUSP) 1 dephosphorylates and inactivates members of the MAPK superfamily, in particular, JNKs, p38α, and p38β MAPKs. It functions as an essential negative regulator of innate immune responses, hence disruption of the *Dusp1* gene renders mice extremely sensitive to a wide variety of experimental inflammatory challenges. The principal mechanisms behind the overexpression of inflammatory mediators by *Dusp1^−/−^* cells are not known. In this study, we use a genetic approach to identify an important mechanism of action of DUSP1, involving the modulation of the activity of the mRNA-destabilizing protein tristetraprolin. This mechanism is key to the control of essential early mediators of inflammation, TNF, CXCL1, and CXCL2, as well as the anti-inflammatory cytokine IL-10. The same mechanism also contributes to the regulation of a large number of transcripts induced by treatment of macrophages with LPS. These findings demonstrate that modulation of the phosphorylation status of tristetraprolin is an important physiological mechanism by which innate immune responses can be controlled.

## Introduction

The three canonical MAPK pathways, that is, the p38 MAPK, JNK, and ERK pathways, are commonly activated downstream of pattern recognition receptors including the TLRs. They play critical roles in the initiation and execution of inflammatory responses of myeloid cells (1) and have long been considered promising targets for treatment of inflammatory pathologies (2). Increasingly potent and selective MAPK inhibitors have emerged from drug discovery programs. In combination with genetic targeting approaches, these compounds have been useful for the identification of mechanisms by which the MAPK pathways control expression of inflammatory mediators. Such mechanisms include the activation of downstream kinases, posttranscriptional regulation via the phosphorylation of RNA-binding factors, the phosphorylation and activation of transcription factors, cross talk with other pathways that control transcription, and various combinations of the above. Despite some speculation (3, 4), it is not yet clear why the evident contribution of MAPK pathways to the inflammatory response has so far proved difficult to translate into beneficial drugs. There is a need for greater understanding of how the MAPK pathways are controlled and of what are the most important mechanisms by which they influence the expression of proinflammatory and anti-inflammatory effectors.

The two members of the ERK family, the three members of the JNK family, and the four members of the p38 MAPK family are all activated via phosphorylation of threonine and tyrosine residues within a Thr–Xxx–Tyr activation motif. The amino acid (Xxx) that separates the two phosphorylation sites is a defining characteristic of each MAPK family. The most energy efficient means of inactivating MAPKs and promoting the termination of an inflammatory response is via removal of the activating phosphate groups. To a large extent, this process is dependent on MAPK phosphatases (MKPs), a subset of a family of enzymes known as dual-specificity phosphatases (DUSPs), because they are able to dephosphorylate both tyrosine- and threonine-phosphate residues (5). Mathematical modeling of MAPK signal transduction pathways suggests that controlling the expression of DUSPs is an important means of determining how cells are able to respond to external stimuli (6, 7).

In mammals, there are believed to be 10 catalytically active MKPs, differing in their substrate specificity, subcellular localization, and pattern of expression (5). The founding member of the family is known as MKP-1 or DUSP1. In resting cells, it is typically expressed at low or undetectable levels, but its expression is rapidly induced by a wide variety of proinflammatory agonists, usually in a p38 MAPK-dependent manner. Although it may target ERK for dephosphorylation and inactivation under some circumstances, DUSP1 displays preference for JNK and p38 MAPK as substrates (8). The p38-dependent expression of an enzyme that inactivates p38 MAPK constitutes a classical negative feedback loop, which is essential for the limitation and termination of inflammatory responses in vitro or in vivo (8–13). *Dusp1^−/−^* mice are healthy and fertile under normal conditions, but their deficit in feedback control renders them susceptible to inflammatory challenges. They are highly sensitive to Gram-negative and -positive sepsis (14–16), LPS-induced bone loss (17, 18), cardiac dysfunction (19), and lethal endotoxemia (20–23). In an experimental model of rheumatoid arthritis, disease penetrance, speed of onset, and severity of symptoms were all increased in *Dusp1^−/−^* mice (24). Experimental colitis (25), anaphylaxis, contact hypersensitivity (26), and TNF-induced systemic inflammation (27) were also exacerbated in the absence of DUSP1. Unsurprisingly, DUSP1 is also targeted by several proinflammatory and anti-inflammatory agonists as a means of modulating the activity of the p38 MAPK pathway (or other MAPK pathways) and influencing the outcome of inflammatory challenges. Glucocorticoids (27–29), vitamin D (30), TGF-β (31), and IL-10 (10, 32) exert anti-inflammatory effects in part by increasing the expression of DUSP1. On the contrary, IFN-γ (33) and IL-17A (34) may prolong p38 MAPK signaling and enhance inflammatory responses via negative regulation of DUSP1.

Dysregulated responses to proinflammatory stimuli and attenuated effects of anti-inflammatory mediators are characteristic of cells or mice lacking DUSP1. Yet it remains unclear exactly how the alterations of MAPK signaling in the absence of DUSP1 lead to increased expression of inflammatory mediators. Studies focusing on small numbers of genes have suggested increases in transcription mediated by AP-1 or NF-κB (23, 35–37), or stabilization of mRNA mediated by the RNA-binding factor AU-rich element binding factor 1 (38). In this study, we use a genetic approach to demonstrate that DUSP1 controls the inflammatory response of macrophages to LPS to a large extent by modulating the activity of the mRNA destabilizing protein tristetraprolin (TTP).

## Materials and Methods

### Materials

LPS (*Escherichia coli* serotype EH100) was purchased from Enzo Life Sciences. Other biochemicals were purchased from Sigma-Aldrich unless otherwise stated. All media and sera were routinely tested for endotoxin using the *Limulus* amebocyte lysate test (Lonza) and were rejected if the endotoxin concentration exceeded 0.1 U/ml.

### Generation of mouse strains

Generation of a *Zfp36aa/aa* mouse strain on C57BL/6 background is described in the companion paper (39). The *Dusp1^−/−^* strain was a generous gift from Bristol-Myers Squibb and was backcrossed to C57BL/6 for 10 generations before experiments described in this study. The double-targeted *Dusp1^−/−^*: *Zfp36aa/aa* line was generated by three generations of crossing, with genotyping performed by PCR of genomic DNA, and then was maintained as a pure-breeding line.

### In vivo experiments and cell isolation

All animal experiments were approved by local ethical committees and performed under U.K. Home Office Project Licenses. C57BL/6 mice were purchased from Harlan Laboratories. All mice used were between 6 and 12 wk of age. To assess the systemic response to LPS, we injected mice i.p. with 5 mg/kg purified LPS in 200 μl sterile PBS. Mice were humanely culled 3 h after challenge, and peripheral blood was collected by cardiac puncture for serum isolation.

Bone marrow was isolated from humanely culled mice, and bone marrow–derived macrophages (BMMs) obtained by differentiation in vitro with 100 ng/ml M-CSF (PeproTech) in RPMI 1640 containing 10% heat-inactivated FCS and penicillin/streptomycin for 7 d. BMMs were plated at a density of 1 × 10^6^/ml in the appropriate cell culture plate at least 1 d before stimulation. Primary peritoneal macrophages were harvested by lavaging the peritoneal cavity with 5 ml PBS containing 2 mM EDTA. Lavage fluid was collected and cells were resuspended at 2 × 10^6^/ml in DMEM supplemented with 10% heat-inactivated FCS and penicillin/streptomycin. Cells were plated and allowed to adhere for 1 h at 37°C, before being washed twice with media. The remaining adherent cells were >90% F4/80+ macrophages as assessed by flow cytometry. Cells were rested overnight before stimulation.

### Assessment of protein expression

Secreted factors in tissue culture supernatants and sera were quantified by ELISA according to manufacturer’s instructions (eBioscience) or by using Bio-Plex bead capture assays and a Bio-Plex 200 analyzer (Bio-Rad). Cell lysates were resolved on SDS-PAGE gels and probed with primary Abs, and immunoreactive proteins were visualized with HRP-coupled secondary Abs and chemiluminescence reagents (Bio-Rad, Pierce, or Cell Signaling Technology). Primary Abs were from Cell Signaling Technology, with the exception of an anti-TTP antiserum that was described previously (40). Blots were visualized using the ChemiDoc MP Imaging System, and in some cases quantified using Image Lab software (Bio-Rad).

### Measurement of mRNA

RNA was extracted from BMMs using QIAshredder columns and RNeasy Mini kit (Qiagen). cDNA was generated using the iScript cDNA Synthesis Kit (Bio-Rad). Gene expression was quantified by quantitative PCR on a LightCycler 480 II (Roche) using Superscript III platinum RT-PCR kit and custom-synthesized oligonucleotide primers (Eurofins MWG) with SYBR Premix Ex Taq (Lonza). Relative gene expression was calculated using the ΔΔ threshold cycle method with *Gapdh* mRNA for normalization of RNA levels. Primary transcript PCR was performed using primer pairs that crossed exon-intron boundaries, with an additional DNaseI step to remove contaminating genomic DNA from RNA samples (Qiagen). Control PCRs were carried out in the absence of RT to monitor genomic DNA contamination, which in all cases was negligible. Sequences of oligonucleotides designed to detect primary or mature transcripts are available on request from the authors.

For microarray analysis, RNA was extracted as described earlier and purified using RNA clean and concentrator kits (Cambridge Bioscience). Microarray hybridization and analysis were performed by Oxford Gene Technology Total RNA or control RNA were converted to labeled cRNA with Cy3 or Cy5, respectively, using the Low Input Quick Amp Labeling Kit, Two-Color (Agilent Technologies). Samples were hybridized onto SurePrint G3 Mouse GE 8 × 60K slides and read using a G2505C scanner (Agilent Technologies). The scanned images were analyzed with Agilent Feature Extraction Software 10.7.3.1 using default parameters. Processed Signal intensities were background subtracted and spatially detrended.

### Statistical analysis

GraphPad Prism software (Version 5.03) was used for statistical analysis. Unpaired, two-tailed Student *t* test was applied for comparison of two groups. For analysis of multiple groups, ANOVA was used with Bonferroni correction for multiple comparisons. Asterisks are used throughout: *p < 0.05, **p < 0.01, ***p < 0.005; n.s. represents not statistically significant.

### Microarray analysis

Probe intensity values for the microarray were analyzed with Partek Genomics Suite version 6.6, build 6.13.0315 (Partek). A two-way mixed-model ANOVA was performed on the entire dataset (55681 probes) to calculate pairwise contrasts consisting of a corrected step-up *p* value (false discovery rate by the Benjamini–Hochberg step-up method integrated into the Partek software) and a fold change or ratio for difference in gene expression. Data from 1- and 4-h LPS treatment were analyzed separately by ANOVA. Weakly expressed transcripts were filtered out if their log-transformed sample intensity values did not exceed an arbitrary value of 100 in at least two of three replicate samples from LPS-treated wild type or *Dusp1^−/−^* BMMs. Transcripts with aberrant outlying sample intensity values (SD of three replicates > mean of three replicates) were also filtered out. The microarray data described in this article have been submitted to the Gene Expression Omnibus’s National Center for Biotechnology Information (GSE68449; http://www.ncbi.nlm.nih.gov/).

## Results

### Increased mRNA stability contributes to the overexpression of cytokines and chemokines by *Dusp1^−/−^* macrophages

**FIGURE 1.**
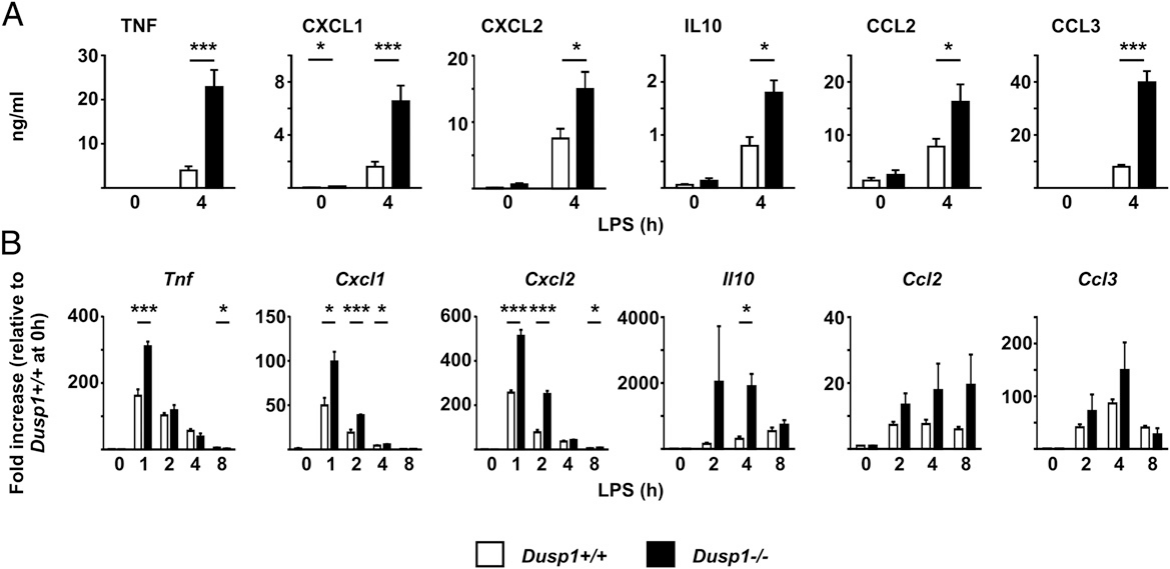
LPS-treated *Dusp1^−/−^* BMMs overexpress cytokine and chemokine mRNA and proteins. (**A**) *Dusp1^+/+^* and *Dusp1^−/−^* BMMs were treated with LPS for 4 h, and secreted cytokines and chemokines in supernatants were measured by multiplex bead capture assay. Graphs represent mean ± SEM of at least three independent experiments. (**B**) *Dusp1^+/+^* and *Dusp1^−/−^* BMMs were treated with LPS for the times indicated and mRNAs were measured by quantitative PCR, with normalization first against *Gapdh* mRNA and then against unstimulated *Dusp1^+/+^* BMMs. Graphs represent means ± SEM from three to four independent experiments. *p < 0.05, ***p < 0.005.

**FIGURE 2.**
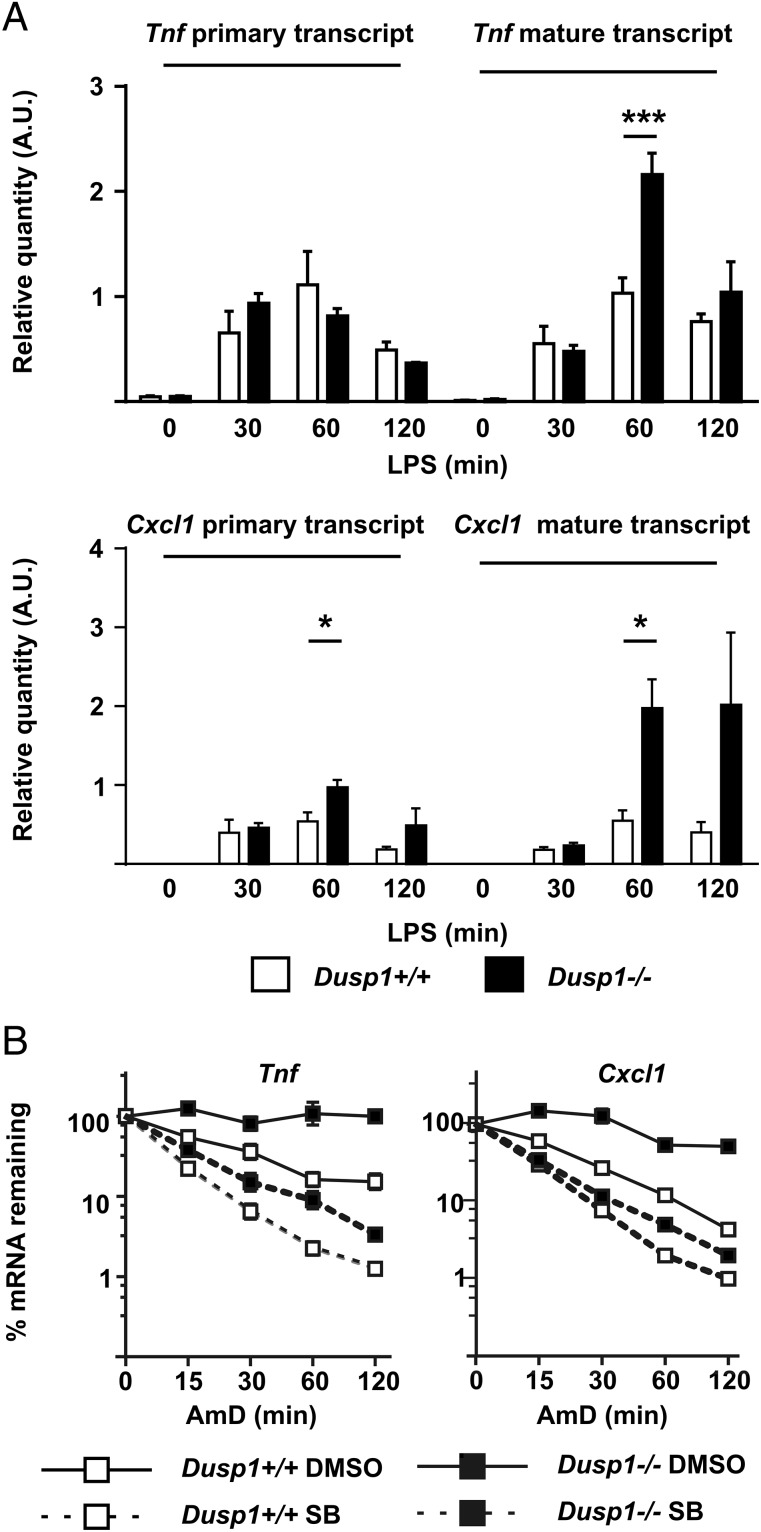
Enhanced stability of *Tnf* and *Cxcl1* mRNAs in *Dusp1^−/−^* BMMs. (**A**) *Dusp1^+/+^* and *Dusp1^−/−^* BMMs were treated with LPS for the indicated times, RNA was harvested, and *Tnf* and *Cxcl1* primary and mature (spliced) mRNAs were measured by quantitative PCR with normalization first against *Gapdh* and then against levels in *Dusp1^−/−^* BMMs treated with LPS for 60 min. Graphs show mean ± SEM of four independent experiments. (**B**) *Dusp1^+/+^* and *Dusp1^−/−^* BMMs were treated with LPS for 1 h before addition of Actinomycin D (10 μg/ml) and DRB (50 μM) to halt transcription, in the presence of either 1 μM SB202190 or vehicle control (0.1% DMSO). RNA was harvested after the times indicated; *Tnf* and *Cxcl1* mRNAs were measured by quantitative PCR with normalization first against *Gapdh* and then against *Tnf* or *Cxcl1* mRNA levels at the appropriate zero time point. Graphs represent mean ± SEM of three independent experiments. Most error bars are obscured by symbols. *p < 0.05, ***p < 0.005.

As previously reported (20–23), LPS-treated *Dusp1^−/−^* BMMs overexpressed a variety of cytokines and chemokines at both protein and mRNA levels (Fig. 1). To identify mechanisms involved in this exaggerated response, we initially focused on *Tnf* and *Cxcl1*. Unspliced primary transcripts were quantified as a means of assessing LPS-induced transcription. No differences of *Tnf* transcription rates were detected between *Dusp1^+/+^* and *Dusp1^−/−^* BMMs, whereas in the same experiments an increase in abundance of mature *Tnf* mRNA was detected in *Dusp1^−/−^* BMMs (Fig. 2A, *upper panel*). One hour after addition of LPS, the levels of *Cxcl1* mature transcript were 3.6-fold higher in *Dusp1^−/−^* than *Dusp1^+/+^* BMMs (*p* < 0.05), whereas primary transcript was only 1.8-fold higher (*p* < 0.05, Fig. 2A, *lower panel*).

Alterations of transcription were not sufficient to account for the strong increases in expression of *Tnf* and *Cxcl1* mRNAs in *Dusp1^−/−^* BMMs. Actinomycin D chase experiments were therefore performed to assess mRNA stability. Both *Tnf* and *Cxcl1* mRNAs were strongly stabilized in *Dusp1^−/−^* BMMs relative to *Dusp1^+/+^* controls (Fig. 2B). In macrophages of both genotypes, a selective p38 MAPK inhibitor increased the rate of degradation of *Tnf* and *Cxcl1* mRNAs. Similar behavior was also observed in the cases of *Cxcl2* and *Il10* mRNAs; they were more stable in *Dusp1^−/−^* than *Dusp1^+/+^* BMMs and were consistently destabilized by inhibition of p38 MAPK (data not shown). These results indicate that p38 MAPK-dependent mRNA stabilization contributes to the dysregulated expression of cytokines and chemokines in macrophages lacking DUSP1.

**FIGURE 3.**
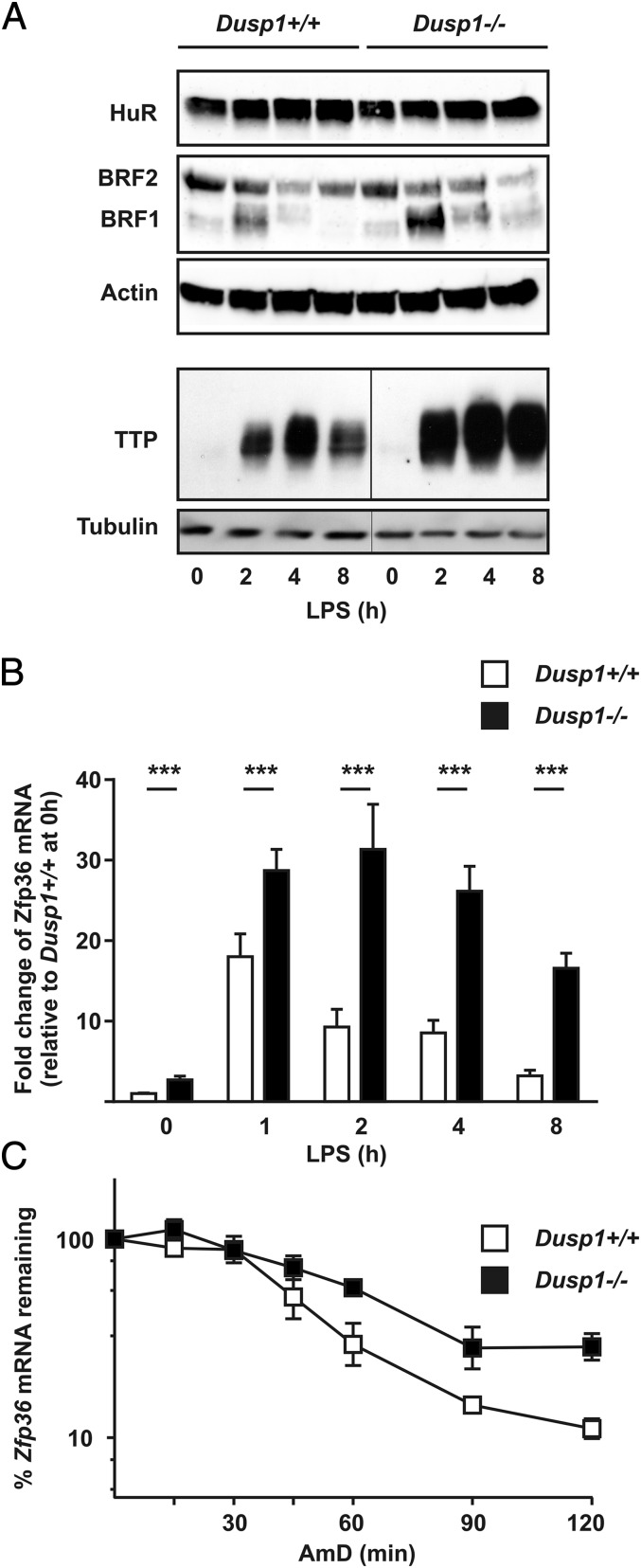
Enhanced expression of TTP in *Dusp1^−/−^* BMMs. (**A**) *Dusp1^+/+^* or *Dusp1^−/−^* BMMs were treated with LPS for the indicated times, whole-cell lysates were prepared, and the ARE-binding proteins HuR, BRF1 and BRF2 were detected by Western blotting, with actin as a loading control. Representative of two experiments. In a separate experiment (*lower two panels*), cells were identically treated and TTP was detected by Western blotting, with tubulin as a loading control. Representative of five experiments. Vertical lines indicate where lanes from a single Western blot exposure have been spliced together. (**B**) *Dusp1^+/+^* or *Dusp1^−/−^* BMMs were treated with LPS for the indicated times, and *Zfp36* mRNA was measured by quantitative PCR with normalization first against *Gapdh* and then against levels in unstimulated *Dusp1^+/+^* BMMs. Graphs represent mean ± SEM of nine independent experiments. (**C**) *Dusp1^+/+^* or *Dusp1^−/−^* BMMs were treated with LPS for 1 h before addition of Actinomycin D (10 μg/ml) and DRB (50 μM) to halt transcription. RNA was harvested at the indicated times and *Zfp36* mRNA was measured by quantitative PCR with normalization first against *Gapdh* and then against levels at the appropriate zero time point. Graph represents mean ± SEM of four independent experiments. ***p < 0.005.

To identify potential mechanisms of posttranscriptional dysregulation, we investigated the expression of selected mRNA binding proteins in *Dusp1^+/+^* and *Dusp1^−/−^* BMMs. HuR is an mRNA stabilizing factor that has been implicated as a mediator of posttranscriptional effects of the p38 MAPK signaling pathway (41–43). TTP and butyrate response factor-1 (BRF-1) and -2 are related mRNA-destabilizing proteins that recognize adenosine/uridine-rich elements (AREs) in the 3′ untranslated regions (UTRs) of target transcripts and recruit components of the cellular mRNA degradation machinery (44, 45). Downstream of p38 MAPK, MK2 phosphorylates and inactivates TTP by reducing its affinity for AREs or preventing the recruitment of mRNA degradation factors, resulting in transient and p38 MAPK-dependent stabilization of target transcripts (46–49). Expression of HuR and BRF-2 did not differ between *Dusp1^+/+^* and *Dusp1^−/−^* BMMs, but levels of both TTP and BRF-1 were higher in the latter (Fig. 3A). The function of BRF-1 is discussed later. Here we focus on TTP, which is encoded by the *Zfp36* gene. Expression of TTP protein was increased by a factor of 1.4 ± 0.03 (*n* = 3) at the 4-h time point in *Dusp1^−/−^* BMMs. Expression of *Zfp36* mRNA was enhanced and prolonged in *Dusp1^−/−^* BMMs (Fig. 3B), and its stability was increased (Fig. 3C).

### Enhanced expression of certain cytokines and chemokines by *Dusp1^−/−^* macrophages is dependent on phosphorylation and inactivation of TTP

TTP autoregulates its expression via interaction with AREs in its own (i.e., *Zfp36*) mRNA (39, 44). It is also well-known to destabilize *Tnf*, *Cxcl1*, *Cxcl2*, and *Il10* mRNAs (44). It therefore seems paradoxical that increased expression of TTP protein is accompanied by increased stability of transcripts targeted for degradation by TTP. However, there is a straightforward resolution to this paradox. It has been previously postulated, and it is conclusively demonstrated in an accompanying article (see Ref. 39), that p38 MAPK-dependent, MK2-mediated phosphorylation of serines 52 and 178 not only inactivates TTP, but also protects TTP from destruction by the proteasome. Activation of p38 MAPK leads to the accumulation of TTP in an inactive form, permitting transient stabilization of target mRNAs and expression of their protein products. We previously created a knock-in mouse strain, in which serines 52 and 178 of endogenous murine TTP were substituted by nonphosphorylatable alanine residues. The mutant *Zfp36aa* allele protected mice against excessive inflammatory responses to i.p. injection of LPS. In macrophages derived from this strain, TTP protein was constitutively degraded by the proteasome and therefore expressed at very low levels. However, it also promoted efficient degradation of target mRNAs because it could not be inactivated. We hypothesized that targeted deletion of *Dusp1* gives rise to an opposite phenotype, in which the unusually prolonged activation of p38 MAPK drives phosphorylation of serines 52 and 178, resulting in accumulation of the inactive form of TTP and the enhanced stabilization of its target mRNAs. To test this hypothesis, we generated a doubly-targeted mouse strain, in which the disruption of the *Dusp1* locus was combined with targeted mutation of the *Zfp36* locus. Our hypothesis predicts that mutation of the *Zfp36* locus will prevent the dysregulated expression of cytokines and chemokines caused by *Dusp1* deletion.

**FIGURE 4.**
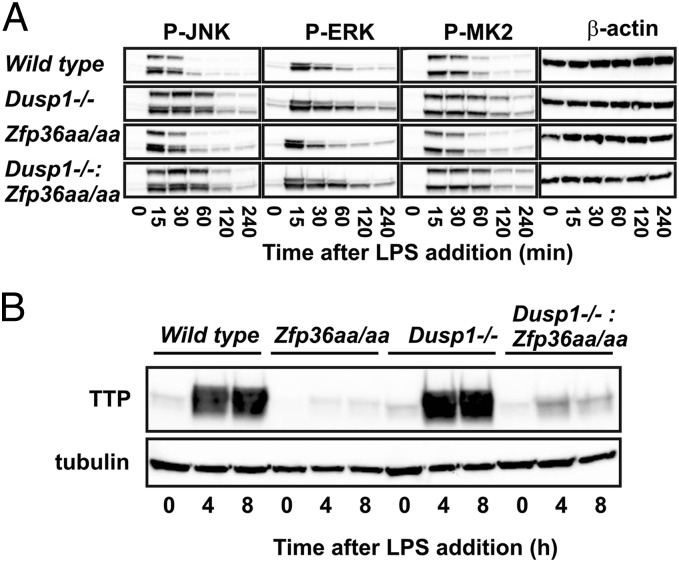
Enhanced expression of TTP in *Dusp1^−/−^* BMMs is dependent on intact phospho-acceptor sites S52 and S178. (**A**) Wild type, *Dusp1^−/−^*, *Zfp36aa/aa*, and *Dusp1^−/−^*: *Zfp36aa/aa* BMMs were treated with LPS for the times indicated; whole-cell lysates were prepared and phosphorylated kinases were detected by Western blotting. Representative of two identical experiments. (**B**) BMMs of the same four phenotypes were stimulated with LPS for 0, 4, or 8 h, lysates were prepared, and TTP protein was detected by Western blotting. Representative of two identical experiments.

BMMs were prepared from mice of four genotypes: wild type (*Dusp1^+/+^*:*Zfp36^+/+^*), *Dusp1^−/−^*, *Zfp36aa/aa*, and *Dusp1^−/−^*:*Zfp36aa/aa*. MAPK signaling responses to LPS were tested (Fig. 4A), and expression of TTP protein was assessed (Fig. 4B) by Western blotting. Phosphorylation of MK2 was measured as a surrogate for p38 MAPK activity, which is particularly relevant because of its link to TTP phosphorylation, stabilization, and inactivation. The activation of all three major MAPK signaling pathways was prolonged in *Dusp1^−/−^* BMMs, reflecting a failure of one important off-mechanism (Fig. 4A). As previously shown (Fig. 3A), expression of TTP protein was elevated in these cells (Fig. 4B). In *Zfp36aa/aa* BMMs, the activation of MAPKs was indistinguishable from that in wild type BMMs, but the expression of TTP protein was markedly decreased, as described in the accompanying article (39). In double-mutant (*Dusp1^−/−^*:*Zfp36aa/aa*) BMMs, the activation of MAPKs was prolonged because of the absence of the phosphatase, but expression of TTP protein remained virtually as low as in *Zfp36aa/aa* BMMs. In other words, the dysregulation of MAPK signaling pathways increased the expression of TTP only if the phosphoacceptor sites S52 and S178 were intact. Together with previously published results (50, 51), this confirms that the p38 MAPK–MK2 signaling pathway stabilizes TTP protein via the phosphorylation of serines 52 and 178.

**FIGURE 5.**
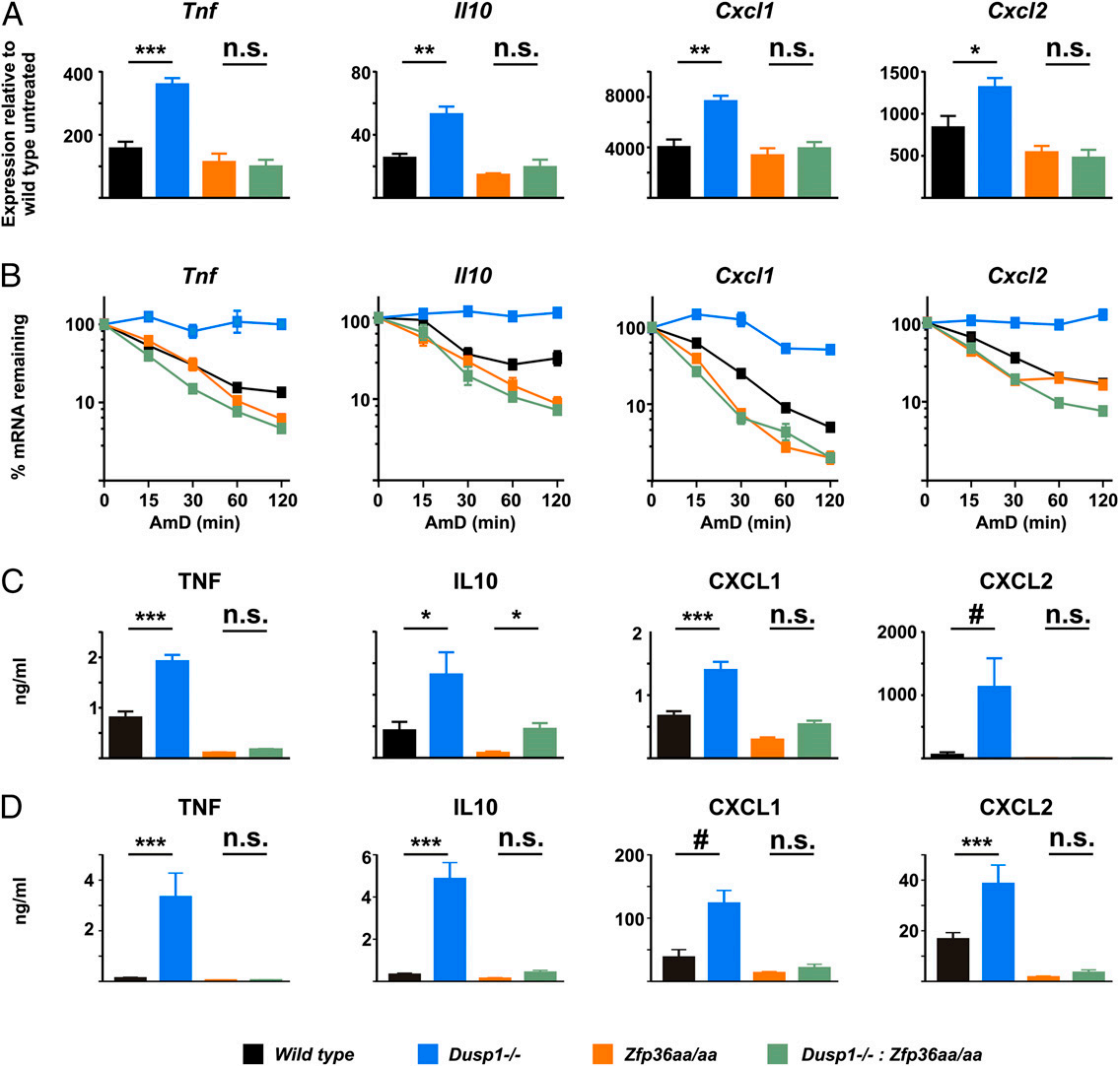
Disruption of the *Dusp1* gene enhances expression of TNF, IL-10, CXCL1, and CXCL2 in a manner that depends upon phosphorylation of TTP. In this and subsequent figures, wild type is indicated by black, *Dusp1^−/−^* by blue, *Zfp36aa/aa* by orange, and *Dusp1^−/−^*: *Zfp36aa/aa* by green. (**A**) Wild type, *Dusp1^−/−^*, *Zfp36aa/aa*, and *Dusp1^−/−^*: *Zfp36aa/aa* BMMs were treated with LPS for 1 h, RNA was harvested, and the indicated transcripts were measured by quantitative PCR with normalization first against *Gapdh* and then against levels in unstimulated wild type BMMs. Graphs represent means ± SEM of three independent experiments. (**B**) BMMs of all four genotypes were treated with LPS for 1 h before addition of Actinomycin D (10 μg/ml) and DRB (50 μM) to halt transcription. RNA was harvested at the indicated times, and the indicated transcripts were measured by quantitative PCR with normalization first against *Gapdh* and then against levels at the appropriate zero time point. Graph represents mean ± SEM of at least three independent experiments in each case. (**C**) BMMs of all four genotypes were treated with LPS for 4 h, and levels of TNF, IL-10, CXCL1, or CXCL2 proteins in supernatants were measured by multiplex bead capture assay. Graphs represent means ± SEM of five independent experiments. In the case of CXCL2, three of five values were outside the linear range of the multiplex assay and were arbitrarily assigned a value equivalent to the highest concentration of the standard curve. (**D**) Mice of all four genotypes were injected i.p. with 10 mg/kg LPS, sacrificed 3 h later, and serum levels of TNF, IL-10, CXCL1, and CXCL2 were measured by multiplex bead capture assay. Graphs represent mean ± SEM of at least seven independent experiments. In the case of CXCL1, five of eight values were outside the linear range of the multiplex assay and were arbitrarily assigned a value equivalent to the highest concentration of the standard curve. ^#^No statistical comparison was performed. *p < 0.05, **p < 0.01, ***p < 0.005. n.s., not statistically significant.

*Tnf*, *Il10*, *Cxcl1*, and *Cxcl2* were all shown to be overexpressed by *Dusp1^−/−^* BMMs, at least in part because of increased mRNA stability. In contrast, the same transcripts were underexpressed by *Zfp36aa/aa* BMMs as a consequence of decreased mRNA stability (49). We asked which phenotype would dominate in macrophages bearing both mutations. In other words, could the dysregulation of MAPK signaling pathways increase the expression of these proinflammatory and anti-inflammatory mediators if it did not result in the phosphorylation of TTP? In a *Zfp36* wild type genetic background, disruption of the *Dusp1* gene significantly increased the expression of *Tnf*, *Il10*, *Cxcl1*, and *Cxcl2* mRNAs as previously described. When both *Zfp36* alleles were mutated, these effects of *Dusp1* gene disruption were absent (Fig. 5A). The half-lives of *Tnf*, *Il10*, *Cxcl1*, and *Cxcl2* mRNAs were increased in *Dusp1^−/−^* BMMs and decreased in *Zfp36aa/aa* BMMs as expected (Fig. 5B). In double-mutant BMMs, the *Zfp36* phenotype dominated. Despite dysregulation of MAPK signaling pathways, *Tnf*, *Il10*, *Cxcl1*, and *Cxcl2* mRNAs remained unstable. At the protein level, similar patterns were observed (Fig. 5C). Expression of TNF, IL-10, CXCL1, and CXCL2 was elevated in LPS-treated *Dusp1^−/−^* BMMs in comparison with wild type BMMs. In contrast, the expression of these cytokines and chemokines was low in *Zfp36aa/aa* BMMs and was not significantly increased by additional disruption of the *Dusp1* locus. Finally, mice of all four genotypes were injected i.p. with LPS, and serum cytokine concentrations were measured after 3 h (Fig. 5D). Expression of TNF, IL-10, CXCL1, and CXCL2 was higher in *Dusp1^−/−^* than wild type mice, but similarly low in *Zfp36aa/aa* and double-mutant mice. In conclusion, the disruption of the *Dusp1* gene and consequent dysregulation of MAPK signaling increased the expression of a set of inflammatory mediators in a manner that absolutely depended on the phosphorylation of TTP serines 52 and 178. Conversely, a normal function of DUSP1 is to promote the destabilization of certain transcripts by altering the equilibrium of TTP phosphorylation and dephosphorylation in favor of the active, dephosphorylated form.

### Involvement of DUSP1 and TTP in the genome-wide response to LPS

To broaden these findings and to determine how extensively DUSP1 controls macrophage responses to LPS via modulation of TTP phosphorylation, we assessed gene expression by microarray in BMMs of all four genotypes after stimulation with LPS for 1 or 4 h. The LPS responses of *Zfp36aa/aa* BMMs were discussed in an accompanying article (39). To our knowledge, there is no published study describing global effects of *Dusp1* gene disruption on the LPS responses of isolated macrophages. A previous study describing gene expression in whole spleens of *Dusp1^−/−^* mice 6 h after i.p. administration of LPS (20) is not directly comparable with ours because of differences of experimental design, statistical treatment, and most importantly, the complexity of cellular populations in the spleen. More than 2000 transcripts were upregulated after treatment of wild type BMMs with LPS for 1 h. Approximately 30% of these transcripts displayed significantly different expression in LPS-treated *Dusp1^−/−^* BMMs, a proportion that changed very little if the transcripts were filtered for increasing strength of induction by LPS (Table I). As expected, the majority of the dysregulated response was related to increases of gene expression in the absence of DUSP1. However, ∼5% of LPS-induced transcripts were underexpressed by *Dusp1^−/−^* BMMs. These included *Irf1*, which was expressed at 2-fold lower levels in *Dusp1^−/−^* BMMs (*p* = 2 × 10^−12^). The *Irf1* gene was previously described as being negatively regulated by p38 MAPK and consequently underexpressed in *Dusp1^−/−^* cells (52). The apparent underexpression of a set of genes in *Dusp1^−/−^* BMMs remains to be confirmed and investigated in detail.

**Table I.** Genome-wide dysregulation of responses to LPS in *Dusp1^−/−^* BMMs

	1 h			4 h		
LPS-Induced Transcripts		↑ in *Dusp1^−/−^*	↓ in *Dusp1^−/−^*		↑ in *Dusp1^−/−^*	↓ in *Dusp1^−/−^*
All	2152	499 (23%)	103 (5%)	5008	1256 (25%)	1183 (24%)
>3-fold	445	106 (24%)	23 (5%)	1479	383 (26%)	298 (20%)
>5-fold	250	56 (22%)	17 (7%)	924	229 (25%)	167 (18%)
>10-fold	143	31 (22%)	6 (4%)	481	112 (23%)	89 (19%)

**FIGURE 6.**
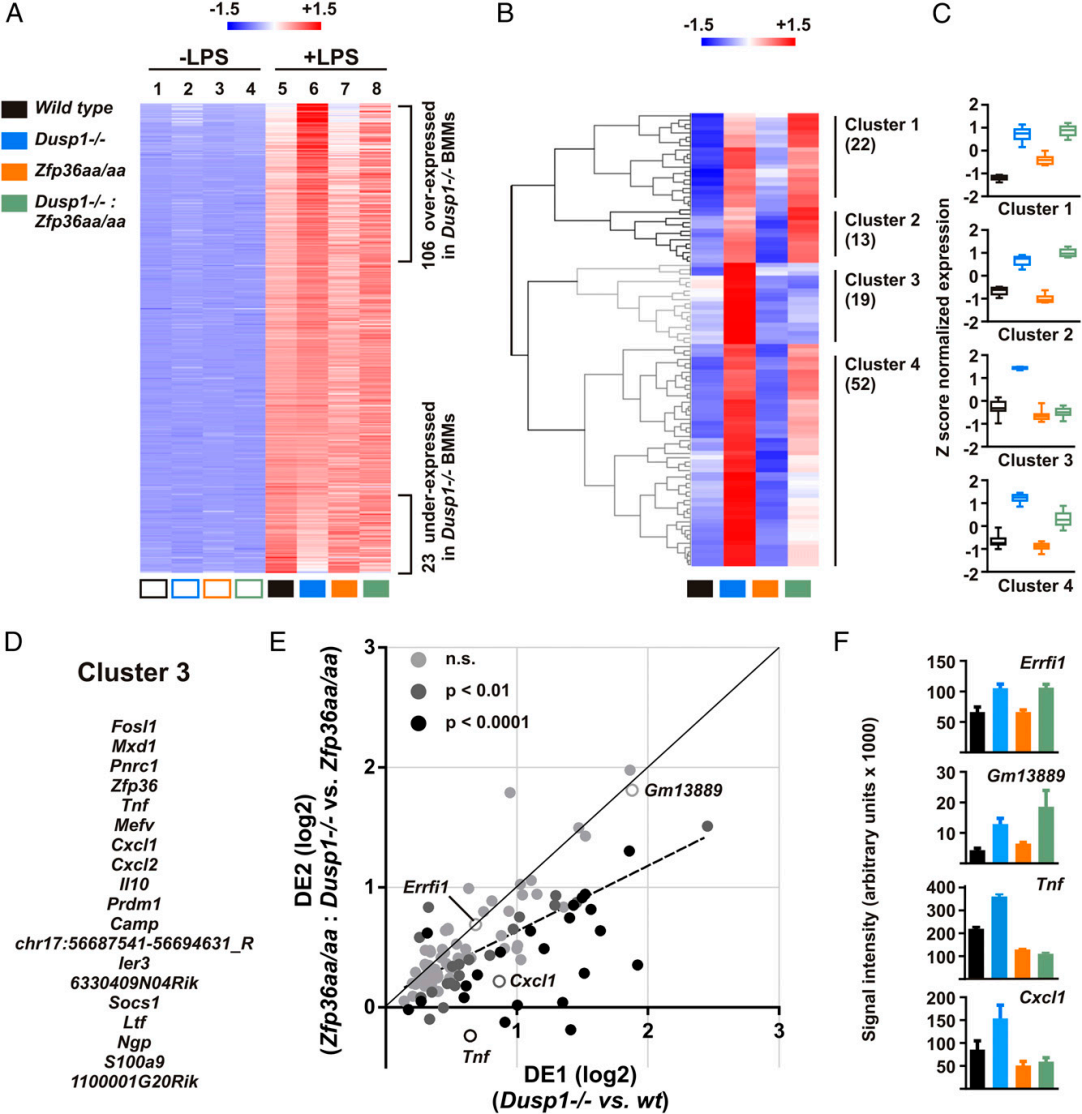
Genome-wide effects of *Dusp1* gene disruption after 1-h LPS are partly dependent on the phosphorylation of TTP. (**A**) BMMs of all four genotypes were left untreated or stimulated with LPS for 1 h and subjected to microarray analysis, using three independent samples for each genotype. The heat map represents transcripts upregulated at least 3-fold by LPS, ranked according to expression in *Dusp1^−/−^* BMMs in comparison with wild type BMMs. Blue represents low and red represents high relative expression. (**B**) The 106 transcripts significantly overexpressed by *Dusp1^−/−^* BMMs were subjected to hierarchical clustering using Genesis. (**C**) Patterns of relative expression within clusters 1–4 are indicated as box and whisker plots. (**D**) Transcripts belonging to cluster 3 are listed. (**E**) For each of the 106 overexpressed transcripts shown in (B), DUSP1 effects DE1 and DE2 were calculated as described in the text and plotted on a log _2_ scale. Error was estimated by pairwise comparison of all three replicate measurements from each genotype, and on this basis a *p* value was assigned for the difference between DE1 and DE2. Light gray points represent individual transcripts for which DE1 and DE2 did not differ significantly; dark gray points represent transcripts for which DE1 and DE2 were significantly different (*p* < 0.01); black points represent transcripts for which DE1 and DE2 were highly significantly different (*p* < 0.0001). The solid diagonal represents the null hypothesis (DE1 = DE2). The dotted line represents the best-fit regression of all data points. (**F**) For selected transcripts, raw expression data from the microarray experiment are plotted as mean ± SEM (*n* = 3).

Microarray data from the four genotypes of macrophage were used to investigate the functional interaction between DUSP1 and TTP in the regulation of macrophage responses to LPS. The heat map in Fig. 6A illustrates 445 transcripts upregulated at least 3-fold by LPS in wild type BMMs (*p* < 0.05), ranked according to their expression in *Dusp1^−/−^* BMMs, with 106 significantly overexpressed transcripts at the top and 23 significantly underexpressed transcripts at the bottom. This pattern of underexpression and overexpression was disrupted by mutation of the *Zfp36aa* locus (compare tracks 6 and 8). Hierarchical clustering of the 106 overexpressed transcripts across all four genotypes (Fig. 6B, 6C) identified two clusters (1, 2), in which disruption of the *Dusp1* gene increased expression whether the *Zfp36* locus was mutated or wild type. Cluster 3 contained 19 transcripts whose expression was increased by *Dusp1* gene disruption in a manner that strongly depended on serines 52 and 178 of TTP (Fig. 6D). These transcripts, including *Cxcl1*, *Cxcl2*, *Ier3*, *Il10*, *Tnf*, and *Zfp36*, appeared to be controlled by DUSP1 exclusively via the phosphorylation of TTP serines 52 and 178. Equally important, there was a large cluster of genes (cluster 4) in which the effects of *Dusp1* gene disruption were impaired rather than completely lost if the two phospho-acceptor sites of TTP were mutated.

These results suggest that DUSP1 regulates more than half of the genome-wide response to LPS wholly or partly via the phosphorylation of TTP. To test this more rigorously, we posed the null hypothesis that disruption of the *Dusp1* gene influences the macrophage response to LPS in a manner that is completely independent of TTP phosphorylation. For each LPS-induced transcript, the effect of disrupting the *Dusp1* locus was calculated as the mean ratio of expression in *Dusp1^−/−^* BMMs to that in *Dusp1^+/+^* BMMs. In the context of a wild type *Zfp36* locus, this DUSP1 effect is referred to as DE1. In the context of a mutated (*Zfp36aa*) locus, the ratio is DE2. According to the null hypothesis, the effect of *Dusp1* gene deletion is the same regardless of genotype at the *Zfp36* locus; that is, DE1 = DE2. In Fig. 6E, DE2 is plotted against DE1 for each of the transcripts shown in Fig. 6B. The null hypothesis is represented by a solid diagonal line with gradient 1.0. Some transcripts (indicated as light gray dots in Fig. 6E) behaved exactly as predicted by the null hypothesis and were very similarly influenced by *Dusp1* gene deletion regardless of *Zfp36* genotype. Two example transcripts, *Gm13889* and *Errfi1*, are indicated as open circles in Fig. 6E, and their patterns of expression are plotted in Fig. 6F. In contrast, many transcripts were more strongly affected by *Dusp1* gene disruption if the *Zfp36* locus was wild type than if it was mutated (i.e., DE1 > DE2). Two examples, *Tnf* and *Cxcl1*, are indicated as open circles in Fig. 6E, and their expression patterns plotted in Fig. 6F. The distribution of data points below the diagonal generated a best fit regression with gradient of 0.55 (95% confidence interval 0.42–0.67), indicated by a dotted line in Fig. 6E, and considerably lower than the value of 1.0 predicted by the null hypothesis. A similar analysis was carried out on all LPS-induced transcripts significantly overexpressed by *Dusp1^+/+^* BMMs, without regard to the magnitude of induction by LPS. This analysis of 499 transcripts gave a very similar pattern, with best fit regression slope of 0.53 (95% confidence interval 0.47–0.59, data not shown). Likewise, the top, middle, and bottom tertiles of transcripts in terms of strength of response to LPS showed the same behavior (data not shown), indicating that DUSP1 controls both weakly and strongly induced transcripts via TTP phosphorylation.

**FIGURE 7.**
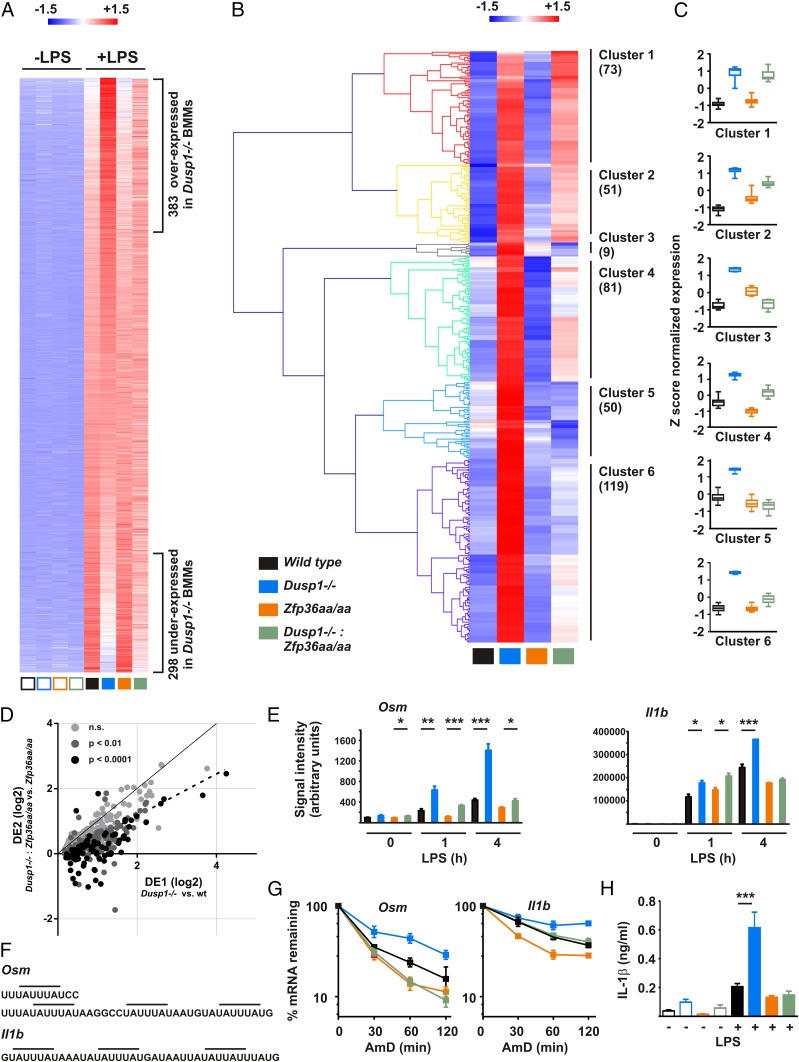
Genome-wide effects of *Dusp1* gene disruption after 4-h LPS are partly dependent on the phosphorylation of TTP. (**A**) BMMs of all four genotypes were left untreated or stimulated with LPS for 4 h and subjected to microarray analysis, using three independent samples for each genotype. The heat map represents transcripts upregulated at least 3-fold by LPS, ranked according to expression in *Dusp1^−/−^* BMMs in comparison with wild type BMMs. (**B**) The 383 transcripts significantly overexpressed by *Dusp1^−/−^* BMMs were subjected to hierarchical clustering using Genesis. (**C**) Patterns of relative expression within clusters 1–6 are indicated as box and whisker plots. (**D**) For each of the 383 overexpressed transcripts shown in (B), DUSP1 effects DE1 and DE2 were calculated and plotted as in Fig. 6. The dotted line represents the best-fit regression of all data points. (**E**) Patterns of expression of *Osm* and *Il1b* mRNA in BMMs of all four genotypes, as determined by microarray. (**F**) AREs in the 3′ UTRs of murine *Osm* and *Il1b* mRNAs. Matches to the consensus TTP binding site are indicated by horizontal bars. (**G**) BMMs of all four genotypes were treated with LPS for 2 h, then Actinomycin D/DRB chases were performed as in Fig. 5. Graphs represent mean normalized mRNA levels from three independent measurements. (**H**) Mice of all four genotypes were injected i.p. with 10 mg/kg LPS, sacrificed 3 h later, and serum levels of IL-1β were measured by multiplex bead capture assay. Graph represents mean ± SEM of at least seven independent experiments. *p < 0.05, **p < 0.01, ***p < 0.005. n.s., not statistically significant.

**FIGURE 8.**
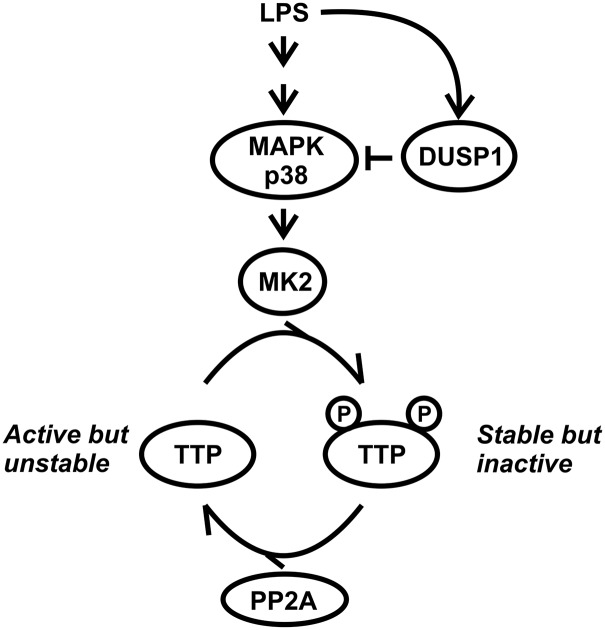
Schematic of the control of TTP function via phosphorylation and dephosphorylation, and the interaction between DUSP1 and TTP.

A total of 5008 transcripts were upregulated after stimulation of wild type macrophages with LPS for 4 h. Forty to 50% of this response was influenced by disruption of the *Dusp1* locus (Table I). Underexpression of genes in *Dusp1^−/−^* BMMs was more evident than at the 1-h time point. Of 1479 transcripts upregulated, at least 3-fold in response to LPS, 383 (26%) were significantly overexpressed and 298 (20%) significantly underexpressed by *Dusp1^−/−^* BMMs (Fig. 7A; Table I). Hierarchical clustering of 383 overexpressed transcripts generated six discrete clusters. Three of these, together containing 178 transcripts, were characterized by impairment, ablation, or reversal of the response to *Dusp1* gene deletion in the *Zfp36aa/aa* background (Fig. 7B, 7C). As in the case of 1-h stimulation, plotting DE2 versus DE1 for each transcript revealed a shift toward the horizontal axis (Fig. 7D), the best fit regression having a slope of 0.64 (95% confidence interval 0.57–0.71). Again this indicates that the consequences of dysregulating LPS-activated MAPK signaling pathways are broadly attenuated by targeted mutation of the *Zfp36* locus. Our analyses of transcript expression across the genome formally demonstrate that DUSP1 regulates the macrophage response to LPS largely (but not exclusively) via the modulation of TTP phosphorylation (Fig. 8). In genetic terms, *Zfp36* is epistatic to *Dusp1*.

It is clearly possible that some of the changes of gene expression described in this study are indirect rather than direct. This applies particularly to the 4-h time point, when there will presumably be greater autocrine or paracrine effects of dysregulated expression of TNF and other factors (10, 53–55). We therefore investigated the stability of a subset of transcripts that appeared to be regulated via the DUSP1–TTP axis at 4 h. At both 1 and 4 h, Oncostatin M (*Osm*) displayed the characteristic pattern of increased expression in *Dusp1^−/−^*, but not *Dusp1^−/−^*:*Zfp36aa/aa* BMMs. This transcript did not appear as a DUSP1–TTP target in the 1-h data set because its fold induction by LPS fell short of the arbitrary 3-fold cutoff (Fig. 7E, *left*). *Il1b* (IL-1β) was strongly upregulated by LPS at both 1 and 4 h, but displayed the signature DUSP1–TTP target pattern of expression only at the later time point (Fig. 7E, *right*). Both of these transcripts contain multiple matches to the consensus TTP binding sequence UAUUUAU in their 3′ UTRs (Fig. 7F). The stability of both transcripts was enhanced in *Dusp1^−/−^*, but not *Dusp1^−/−^: Zfp36aa/aa* BMMs (Fig. 7G), confirming that their decay is regulated by DUSP1 via phosphorylation of TTP serines 52 and 178. *IL1b* was previously identified as a target of TTP (56), but to our knowledge *Osm* was not. Some other transcripts (e.g., *Plaur* and *Socs1*) were increased in *Dusp1^−/−^*, but not *Dusp1^−/−^: Zfp36aa/aa*, BMMs at 4 h (GSE68449; Supplemental Table I), yet did not display corresponding differences of mRNA stability (data not shown). It is likely that the dysregulation of these transcripts is an indirect consequence of the altered expression of TNF, or some other factor that feeds back to influence gene expression in BMMs. Because IL-1β has a well-established role in endotoxic shock (57), we went back to test its expression in sera of LPS-treated mice of all four genotypes. The predicted pattern of overexpression in *Dusp1^−/−^*, but not *Dusp1^−/−^: Zfp36aa/aa*, mice was found (Fig. 7G). IL-1β was not detected in supernatants of LPS-treated BMMs, presumably because of insufficient inflammasome activation under these conditions (data not shown).

## Discussion

DUSP1 is a critical negative regulator of pathological inflammatory responses in experimental models of sepsis or endotoxic shock (8, 12–16, 20–23). Our data demonstrate that DUSP1 limits inflammatory responses to a large extent by controlling the activity state of the mRNA destabilizing protein TTP (Fig. 8). Disruption of the *Dusp1* gene led to increases in the expression of inflammatory mediators, many of which could be prevented by targeted mutation of two sites of phosphorylation of TTP. Inflammatory mediators controlled by this mechanism included TNF, CXCL1, CXCL2, and IL-1β, all of which have well-established roles in lethal systemic responses to LPS (57–60). In vitro experiments using primary macrophages demonstrated that DUSP1 controls the stability of proinflammatory mRNAs via modulation of TTP activity. In vivo experiments using wild type, *Dusp1^−/−^*, *Zfp36aa/aa*, and *Dusp1^−/−^; Zfp36aa/aa* mice showed the physiological relevance of this mechanism in the context of an acute inflammatory response to i.p. LPS injection. For example, after i.p. injection of LPS, serum levels of TNF were ∼50-fold higher in *Dusp1^−/−^* than in *Dusp1^−/−^: Zfp36aa/aa* mice.

Equivalent experiments in more complex and long-term disease models have proved difficult because of the exaggerated inflammatory responses of *Dusp1^−/−^* mice. For example, we attempted to induce arthritis by injection of arthritogenic K/BxN serum into mice of all four genotypes. The *Dusp1^−/−^* mice developed a severe, systemic inflammatory response within 3 d and had to be culled on humane grounds. In contrast, *Dusp1^−/−^: Zfp36aa/aa* mice developed and then resolved arthritis over 22 d, without evidence of systemic inflammation (data not shown). These incomplete observations illustrate the central role of DUSP1 in limiting inflammatory responses, and the difficulty of investigating mechanisms directly in the *Dusp1^−/−^* mouse. They also support the contention that the hyperinflammatory phenotype of the *Dusp1^−/−^* mouse is largely a consequence of inactivation of TTP. The functional cooperation between DUSP1 and TTP is illustrated schematically in Fig. 8. The induction of DUSP1 expression in response to a proinflammatory stimulus constitutes a negative feedback loop, assisting in the deactivation of MAPK p38. The DUSP1-mediated decline in MAPK p38 activity results in a shift in the equilibrium from phosphorylated (inactive) to dephosphorylated (active) TTP. The consequent increase in the mRNA destabilizing activity of TTP drives the off-phase of expression of inflammatory mediators.

It has been previously reported that *Dusp1^−/−^* macrophages overexpress IL-10, as well as proinflammatory cytokines (23). We show in this study that *Il10* mRNA is subject to exactly the same DUSP1–TTP-mediated regulation as the proinflammatory transcripts *Tnf*, *Cxcl1*, *Cxcl2*, and *Il-1b*. This illustrates the point that the initiation and resolution of inflammation are often mechanistically coupled; in the words of Serhan and Savill, “The beginning programs the end” (61). Nevertheless, the overexpression of IL-10 in the absence of DUSP1 is insufficient to prevent excessive inflammatory responses in vivo, perhaps because of its delayed production, or simply because the proinflammatory drive becomes overwhelming (23).

According to microarray data, up to 50% of the enhanced response of *Dusp1^−/−^* macrophages to LPS was significantly impaired by mutation of TTP phospho-acceptor sites. These data should prove a useful starting point for the identification of novel targets of the DUSP1–TTP regulatory access. As an example, we showed that *Osm* mRNA was regulated by DUSP1 via the phosphorylation of TTP, although it remains to be confirmed that its protein product is regulated in the same manner. *Osm* encodes Oncostatin M, a member of the IL-6 family that has proinflammatory functions and has been implicated in pathogenesis of rheumatoid arthritis and other inflammatory conditions (62). However, some caution needs to be applied to the analysis of microarray data because of the potential for indirect autocrine or paracrine effects (10, 53–55). The transcripts *Plaur* and *Socs1* were overexpressed by *Dusp1^−/−^*, but not *Dusp1^−/−^ ; Zfp36aa/aa*, BMMs, yet there was no evidence of differences in mRNA decay. *Plaur* mRNA decayed equally slowly, whereas *Socs1* mRNA decayed equally rapidly in BMMs of all four genotypes (data not shown). Neither of these transcripts contains a canonical UAUUUAU binding site for TTP. We therefore suspect that the dysregulation of these genes is a secondary phenomenon. It may be wisest to combine microarray data mining with analysis of mRNA sequences of putative targets or with RNA immunoprecipitation assays (44).

Clearly, not all of the enhanced LPS responses of *Dusp1^−/−^* macrophages are dependent on altered TTP function. Increased mRNA expression may be partly due to enhanced transcription, as has been suggested by others (23, 35–37). It was also noted that expression of the TTP family member BRF-1 was elevated in LPS-treated *Dusp1^−/−^* macrophages. The genes encoding TTP and BRF-1 have diverged since a duplication at least 400 million years ago. One site of MK2-mediated phosphorylation remains conserved in BRF-1 and has been shown to mediate modulation of BRF-1 function by the MAPK p38-MK2 signaling cascade (63). Whether phosphorylation and inactivation of BRF-1 contributes to the *Dusp1^−/−^* phenotype cannot yet be directly tested.

Results presented in this article confirm the hypothesis (50, 51) that phosphorylation of serines 52 and 178 protects TTP from degradation by the proteasome. Dysregulated MAPK p38 signaling in *Dusp1^−/−^* macrophages increased the expression of TTP protein only if those phosphorylation sites were intact. It was recently reported that unstructured domains of TTP promote its recognition and destruction by the proteasome (64). Perhaps phosphorylation prevents this process by imposing structure, for example, via interaction of phosphorylated TTP with 14-3-3 proteins (44). A consequence of coupled stabilization and inactivation is that the activity and the expression of TTP are inversely related. For example, TTP protein levels were high but activity was low in *Dusp1^−/−^* macrophages, whereas TTP protein levels were low but activity was high in either *Zfp36aa/aa* or *Dusp1^−/−^*: *Zfp36aa/aa* macrophages. In other words, TTP may be most evident where it is least active and most active where it is least evident. It follows that when strong expression of TTP is found at sites of inflammation in vivo, this may be symptomatic of a resolution process that is failing rather than an active, ongoing downregulation of inflammatory mediators. Strong expression of TTP has been described in atherosclerotic plaques (65) and rheumatoid synovia (66). We hypothesize that in these contexts, TTP is predominantly phosphorylated at serines 52 and 178 and therefore inactive, and furthermore that its inactivation contributes to the chronically inflamed state.

In summary, in this study, we have used genetic tools to identify a pathway that tightly regulates expression of a set of key proinflammatory and anti-inflammatory mediators in primary macrophages, and contributes to the regulation of many more mediators of the inflammatory response. The principal function of this pathway is to promote the off phase of the macrophage response to a proinflammatory stimulus, and presumably to assist in resolution of inflammation. We speculate that defects in the function of this pathway could contribute to chronicity of inflammatory disease. Furthermore, we speculate that therapeutic targeting of the DUSP1–TTP pathway might be used to exert anti-inflammatory effects.

## Supplementary Material

Data Supplement
